# Correlation between IL36α and IL17 and Activity of the Disease in Selected Autoimmune Blistering Diseases

**DOI:** 10.1155/2017/8980534

**Published:** 2017-05-10

**Authors:** Agnieszka Żebrowska, Anna Woźniacka, Katarzyna Juczyńska, Kamila Ociepa, Elżbieta Waszczykowska, Izabela Szymczak, Rafał Pawliczak

**Affiliations:** ^1^Department of Dermatology and Venereology, Medical University of Lodz, Lodz, Poland; ^2^Department of Immunopathology, Chair of Allergy, Immunology and Dermatology, Medical University of Lodz, Lodz, Poland

## Abstract

Dermatitis herpetiformis (DH), bullous pemphigoid (BP), and pemphigus vulgaris (PV) are autoimmune bullous skin conditions with eosinophilic and neutrophilic infiltrations. While cytokines are crucial for the affinity and activation of different leukocyte cells in the inflammation and blister formation, there are no studies concerning a role of IL-36. The goal of the study was to analyze whether interleukin 36 is involved in pathogenesis of DH, BP, and PV. And the second aim of the study was the estimation of correlation between Il-36 and IL-17 and titers of specific antibodies in these diseases. Expression of IL-36 and IL-17 was detected in serum in all DH, BP, and PV samples. Serum levels of IL-36 and IL-17*α* were statistically higher in DH, BP, and PV groups as compared to the control group. IL-36*α* levels were statistically higher in DH patients, as compared to patients with PV and BP. Our results showed that IL-36 may be helpful in the diagnostic and monitoring of the activity of the disease. IL 36 may play a relevant role of enrolling eosinophils and neutrophils in DH, BP, and PV and finally provoke tissue injury.

## 1. Introduction

Interleukin (IL) 36 (*α*, *β*, *γ*) and IL-36Ra, called IL36 cytokines, are recent members of the IL1 family of cytokines [1] engaged in the evolution of diseases such as psoriasis [2, 3]. Recent studies settled biochemical properties of Il-36 and their influence on immune cells [1, 4]. In psoriasis, IL-36 appears to exert a proinflammatory action by mediating the crosstalk among dendritic and another skin cells, the enrollment of inflammation cells, and the increase of *γδ* T cells [5]. Ciccia et al. [6] confirmed that *γδ* T cells highly signify the IL-36R and generate elevated levels of IL-36 and IL-17. And this is a proof of a proinflammatory role of IL17+ IL36+ *γδ* T lymphocytes in the immunological responses of the primary Sjogren's syndrome.

There is no literature data that revealed the role of IL-36 in the pathogenesis of autoimmune blistering diseases.

Little is known about the expression pattern of IL-36. Expression of IL36*α* is restricted, whereas the IL-36 receptor appears to be more widely expressed. High expression of IL-36*α* messenger RNA was particularly found in epithelial cells and keratinocytes. In addition, initial studies demonstrated that IL-36*α* mRNA is expressed in leukocytes [7]. In contrast, IL36R is primarily expressed by stromal cells, such as fibroblasts, as well as by keratinocytes [1, 8–10]. Recent data also suggested a role of interleukin-36 and receptor for this cytokine in dendritic cell and T cell interaction. IL-36*α* signals via a complex of the IL1 receptor accessory protein and IL-36R [8–10]. Towne et al. [11] demonstrated that IL-36*α* activates NF-*κ*B via MAPKs and signals through binding to the IL-36R. In vitro assays showed that the proinflammatory stimulus of IL-36*α* was antagonized by IL-36Ra.

The objective of the study was to examine serum levels of IL36, in the pathogenesis of dermatitis herpetiformis (DH), bullous pemphigoid (BP), and pemphigus vulgaris (PV). Comprehension of the role of this interleukin in mechanisms of the formation of skin lesion and correlations between IL-36 and disease's activity may contribute to the evolution of novel therapeutic opportunities in DH, BP, and PV. The aim of the study was to answer the question can IL-36 be used as an index of illness activity and reply to therapy. And we would like to estimate whether IL-36 correlates with the activity of IL-17 and antibody titers.

Dermatitis herpetiformis is a subepidermal blistering disorder with autoimmune origin. The main feature of this disease is both skin and gut lesions. Clinical picture contains polymorphic lesions with strong itching. Gut changes are featured by the villous atrophy. Usually, enteric lesions are without clinical manifestation. The direct immunofluorescence (DIF) test is essential for the diagnosis of this disease. The presence of IgA deposits in the papillae as well as anti-endomysium and anti-tissue and anti-epidermal transglutaminase circulating antibodies (IgA class) is key features for the diagnosis of dermatitis herpetiformis. The influx of neutrophils is the main feature in microscopic view of changed skin. It leads to basement membrane zone protein damages. Devastation of type IV collagen, laminin, and entactin leads to demotion of anchoring fibers and appearance of bullae [12].

The last papers indicated that transglutaminase (TG) is a major DH autoantigen. There are two subtypes of TG: tissue and epidermal [13, 14]. There are deposits of complexes of anti-tTG IgA and TG in skin papillae. They provoke an activation of complement followed by the infiltration of neutrophils, deliverance of proinflammatory cytokines [15, 16], and rise of metalloproteinase production [17].

Another autoimmune bullous disease, with neutrophilic and eosinophilic infiltrate in the skin and linear IgG and C3 deposits along the basement membrane zone, is bullous pemphigoid (BP). A complex of IgG antibodies and antigens found in the basement membrane zone initiate a number of immunological and enzymatic occurrences, causing damage of BMZ proteins and bulla formation [18].

Autoantigens in BP are proteins BPAG1 and BPAG2. BPAG1 (230 kD), proteins connecting the parts of the cytoskeleton with hemidesmosomes and desmosomes. The main antigen in BP is BPAG2 (180 kD collagen XVII) [19]. It is the most important structure responsible for adherence of epidermis to the basement membrane, especially extracellular fragment of BPAG2 with a COOH-terminal collagenous domain. The NC16a part of the main antigen, situated within its extracellular domain, is the most immunogenic fragment of BPAG2 [20–22].

Pemphigus is a severe disease, histologically characterized by intraepidermal blister and acantholysis and immunologically by the presence of circulating and in vivo-bound IgG autoantibodies directed against cell surface antigens of epidermal cells [23].

There are two major variants, pemphigus vulgaris (PV) and pemphigus foliaceus (PF). The clinical distinction among them is determined by the kind of anti-desmoglein (anti-Dsg) antibodies. In PV patients with mucosal predominant character, autoantibodies are mostly directed against Dsg 3, whereas in a mucocutaneous type, anti-Dsg 3 and anti-Dsg 1 autoantibodies are found. In PF, only antibodies against desmoglein 1 are detected [23, 24].

## 2. Material and Methods

### 2.1. Patients

The examined group involved 73 patients, including 25 patients with BP (15 women and 10 men, age range: 58 to 87 years, mean age: 72.5 years) and 25 patients with DH (17 women and 8 men, age range: 18 to 65 years, mean age 45.8 years), and 23 patients with PV (18 women and 5 men, age range: 48 to 79 years, mean age 58.6 years) in the active stage of the disease. Twenty healthy subjects (12 women and 8 men, age range: 40–80 years, mean age 61.6) were enrolled to the control group.

All DH, BP, and PV patients signed informed consent, and the study protocol (#RNN/93/03/KE) was accepted by the Local Ethical Committee of Medical University of Lodz. From all patients in the study group and the healthy control group, the blood was collected to several samples. In all patients, the blood was collected during the active stage of the disease without any administered treatment. Biopsies from skin lesions for histopathological examination and from perilesional skin for immunopathological findings were taken in the study groups.

DIF tests confirmed the presence of deposits of IgA in the top of skin papillae in all the DH patients. IIF tests were positive for IgAEmA (titer 1 : 40–1 : 640, median 1 : 40) in all the DH groups too. Anti-tTG antibodies were present in 20/25 patients (median 8.1 IU/mL; range: 0.0–186.3 IU/mL).

The BP group was at an active phase of the illness, and the average score on the BPDAI (Bullous Pemphigoid Disease Activity Index) was 39 ± 16. According to Ackerman, the histopathologic examinations showed completely developed skin lesions in all BP patients.

In all BP patients, DIF indicated IgG and/or C3 linear deposits along the basement membrane zone. Salt split test showed deposits in the roof of the artificial blister. Using the IIF method, circulating IgG antibodies were confirmed in 22/25 patients, in titers from 1 : 80 to 320 (median 160). All PV patients were at an active phase of the disorder, all with mucocutaneous type. In 23/23 patients, we found circulating pemphigus autoantibodies by IIF, in titers from 1 : 40 to 320 (median 160). Direct immunofluorescence tests showed the deposits of IgG—intercellular space staining pattern.

## 3. Methods

IL-36 and IL-17 levels were examined in serum in all patients. IL-36 and IL-17 were measured by ELISA (Sigma-Aldrich and R&D, United Kingdom). Pemphigus autoantibodies and anti-basement membrane autoantibodies were detected by IIF on the monkey esophagus (EUROIMMUN™). In a positive sample, particular antibodies in the diluted serum sample join to the antigens connected to a constant stage. In the next phase, the fastened antibodies were dyed with fluorescein-labeled immunoglobulins and estimated under a fluorescence microscope. Positive tests can be titrated in steps, and the typical titration compartment is provided by the factor 3.162 (square root of 10).

### 3.1. Statistical Data Analysis

The data were shown as a mean ± standard error. The distributions of all variables were proved normal by Shapiro-Wilk's test. Student's *t*-test was performed to assess the statistical significance of the obtained results. Dataset comparisons, where *p* < 0.05 was recognized statistically significant. The direction and strength of linear correlation between the studied variables were assessed with Spearman correlation coefficient (*r*). *p* value less than 0.05 was recognized statistically significant. Data are shown as mean ± standard error of mean (SEM).

## 4. Results

**Figure 1:**
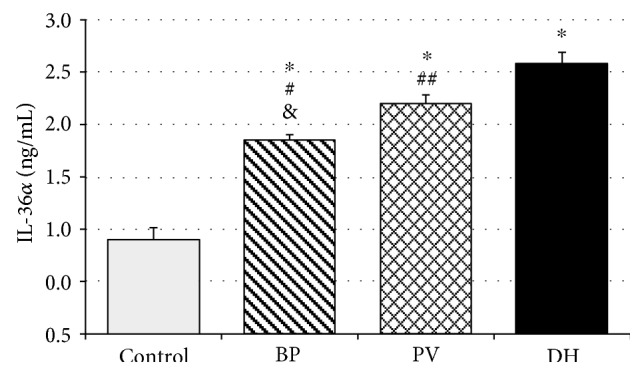
IL-36*α* expression in patients with bullous pemphigoid (BP), pemphigus vulgaris (PV), and dermatitis herpetiformis (DH). Data represent the mean ± SEM. ^∗^*p* < 0.0001 versus that in the control. ^#^*p* < 0.0001; ^##^*p* < 0.02 versus that in DH. ^&^*p* < 0.001 versus that in *p*.

IL-36 serum concentration was statistically significantly higher in DH patients than in PV patients (2.57 ± 0.12 ng/mL versus 2.20 ± 0.08 ng/mL; *p* < 0.05). Patients with BP revealed lower IL-36 (1.85 ± 0.05 ng/mL) concentrations as compared with both DH and PV patients (*p* < 0.02). Interestingly, IL-36 serum concentrations were significantly higher in all the studied patient groups than in the control group, where mean IL36 concentration was 0.89 ± 0.12 ng/mL (*p* < 0.005) Figure 1.

**Figure 2:**
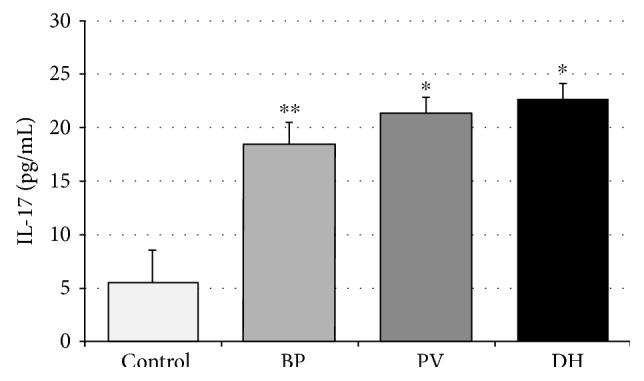
IL-17 expression in patients with bullous pemphigoid (BP), pemphigus vulgaris (PV), and dermatitis herpetiformis (DH). Data represent the mean ± SEM. ^∗^*p* < 0.0001 versus that in the control; ^∗∗^*p* < 0.001 versus that in the control.

IL-17 serum concentration was statistically significantly higher in the DH group than in PV patients (22.76 ± 1.39 ng/mL versus 21.55 ± 1.34 ng/mL; *p* < 0.05). Patients with BP revealed lower IL-17 (18.47 ± 2.07 ng/mL) concentrations as compared with both DH and PV patients (*p* < 0.05). Interestingly, IL-17 serum concentrations were significantly higher in all the studied patient groups as compared to the control group, where mean IL-17 concentration was 5.48 ± 3.11 ng/mL (*p* < 0.0001, *p* < 0.001) Figure 2.

### 4.1. Correlation between IL36*α* and IL17

**Figure 3:**
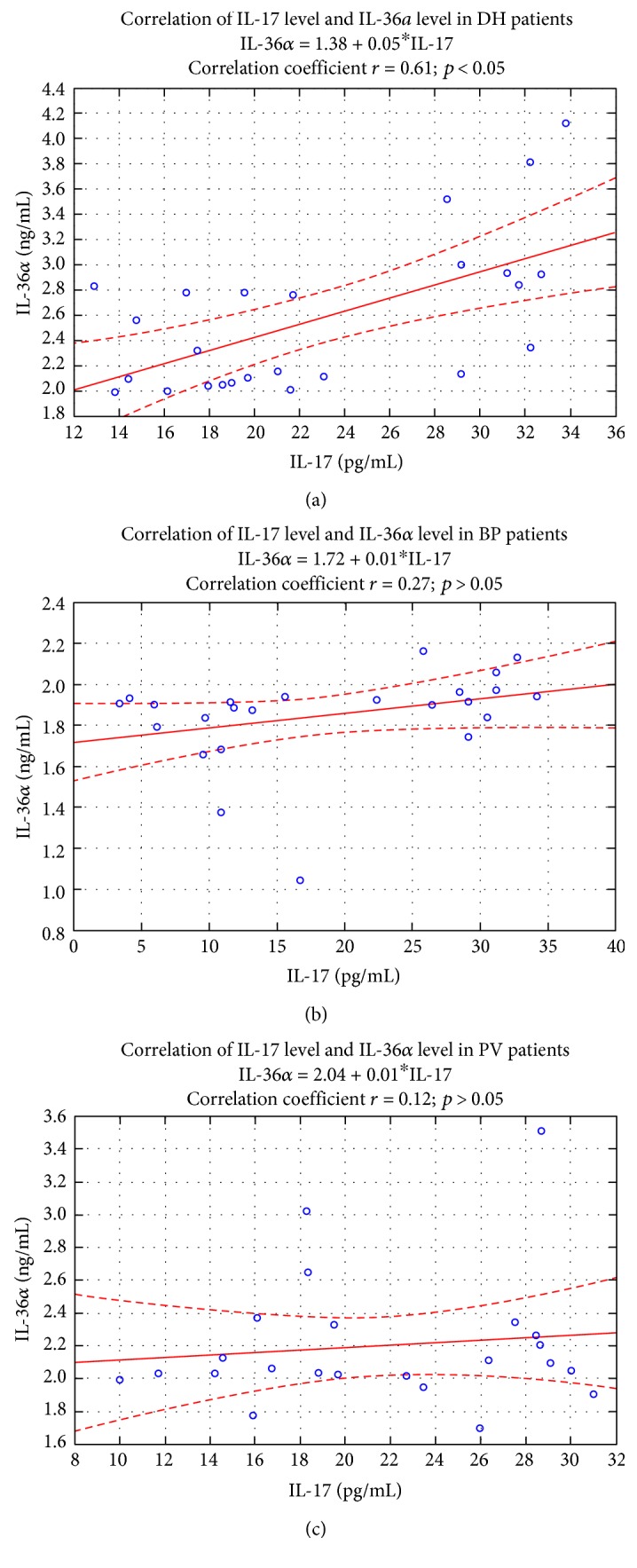
Correlation between IL-17 level and IL-36*α* level in DH patients (*n* = 25). Spearman *r* and *p* are displayed in graph. No correlation was detected between IL-17 level and IL-36A level (*r* = 0.27; *p* > 0.05 for BP patients) (*r* = 0.12; *p* > 0.05 for PV patients).

A positive correlation between IL-17 and IL-36*α* concentrations in the DH group was observed. The correlation factor between IL-17 level and IL-36*α* level was *r* = 0.61 (*p* < 0.05) Figure 3.

### 4.2. Correlation between IL36*α* and Antibody Titers

**Figure 4:**
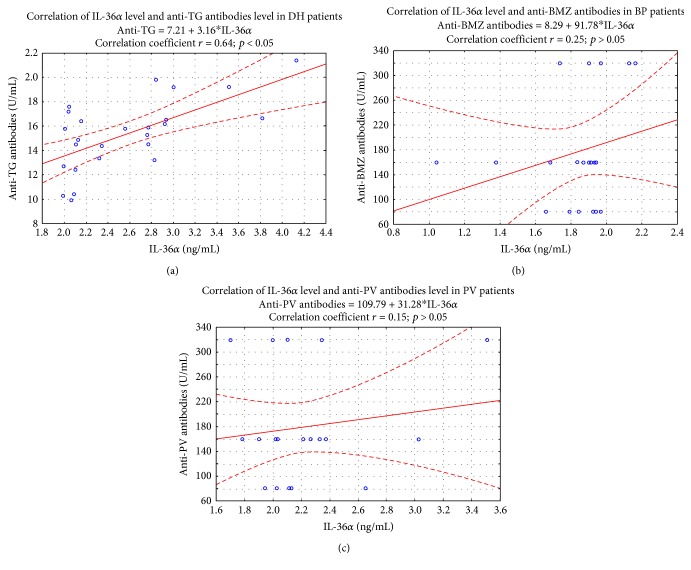
Correlation between IL-36*α* level and anti-TG antibody concentration in DH patients (*n* = 25). Spearman *r* and *p* values are displayed in graph. No correlation was detected between IL-36*α* level and anti-BMZ as well as anti-PV antibodies (*r* = 0.25; *p* > 0.05 for BP patients) (*r* = 0.15; *p* > 0.05 for PV patients).

A positive correlation between IL-36*α* level and concentration of anti-TG antibodies in DH patients was observed. The correlation coefficient between IL-36*α* level and anti-TG antibody concentration was *r* = 0.64 (*p* < 0.05) Figure 4.

### 4.3. Correlation between IL-17 and Antibody Titers

**Figure 5:**
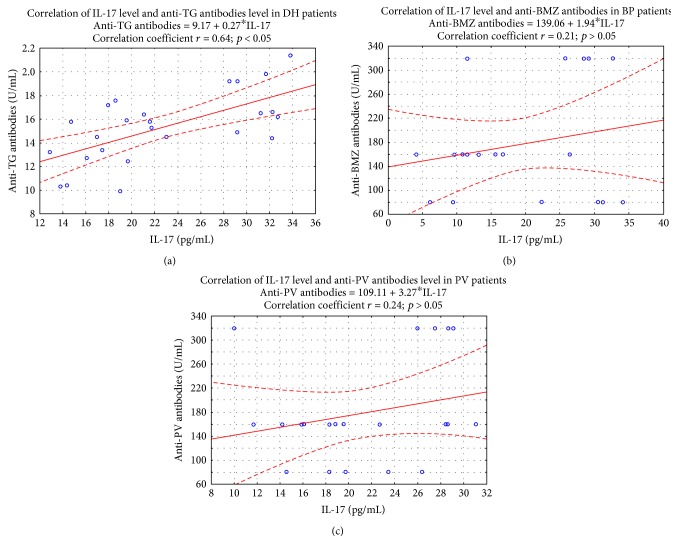
Correlation between IL-17 level and anti-TG antibody concentration level in DH patients (*n* = 25). Spearman *r* and *p* are displayed in graph. No correlation was detected between IL-17 level and anti-BMZ as well anti-PV antibodies (*r* = 0.25; *p* > 0.05 for BP patients) (*r* = 0.24; *p* > 0.05 for PV patients).

A positive correlation betwixt IL-17 level and concentration of anti-TG antibodies in DH patients was observed. The correlation coefficient between IL-17 level and anti-TG antibody concentration was *r* = 0.64 (*p* < 0.05) Figure 5.

## 5. Discussion

Infiltrate in BP and DH is folded of an addition of neutrophils, eosinophils, lymphocytes, and plasma cells. But the acute cells are the major cell populations. According to many studies, the blistering lesions change in time, with the occurrences from mild to severe influx. The reason of these changes is the cascade of inflammatory incidents that appear in sequence [25].

In DH, the influx is consisted of neutrophils in the blister fluid, while in BP, bullae; the infiltrate is organized from mixed cells: neutrophils, eosinophils, lymphocytes, and histiocytes. Ambach et al. [26] examined bullous pemphigoid versus control samples and proved the lack of B cells, suggesting that only antibodies are not able of provoking bulla formation.

The histological examination in pemphigus is the manifestation of intracellular oedema between epidermal cells that finally leads to a disconnection between cells causing an occurrence that we name acantholysis. In the dermis can be found commonly mild to scarce perivascular influx of monocytes and eosinophilic cells. Blister formation provoked by antibodies without inflammation cells is a difference between pemphigus and subepidermal blistering diseases [27].

Ultrastructural researches showed the presence of intense influx at dermo-epidermal connection and damage of BMZ and ingredients of extracellular matrix in BP and DH [18, 19, 21].

Creation of the infiltrates is antedated by accumulation of T cells, regulated by adhesins. The binding of antibodies provokes migration of epidermal cells, production of IL-6 and IL-8, and activation of complement and metalloproteinases in the BMZ [20, 22]. Chemokines are important chemoattractants for both eosinophils and neutrophils [23, 24]. Rare available researches suggest cytokines' role in formation of influx in autoimmune bullous diseases [28–32].

There are data that Th17 cells release cytokines such as interleukin 17 regulators of immunological reactions in various Th1-/Th2-mediated autoimmune complaints like SLE, rheumatoid arthritis, and asthma [33, 34]. There is furthermore suggestion that Th17 cells may take part in the development of blistering skin diseases.

The IL-1 group of cytokines is valid in the first step and support autoimmune responses, and it is engaged in generation of interleukins, matrix enzymes, inflammation cells, all of which are significant agents in pathogenesis of PV, BP, and DH [35–37].

To our best knowledge, there was no data about IL-36 serum levels in blistering diseases and no literature data with reference to the role of IL-36 in development of BP, DH, and PV. The hypothesis is that IL-36 might play a valid role in autoimmune blistering diseases.

Importantly, IL-36*α* has proinflammatory properties and appears that it has a significant role in the development of psoriasis [2, 3]. Patients with a functional deficiency in the *IL-36Rn* gene encoding for the antagonist IL-36Ra suffer from severe generalized pustular psoriasis [38]. IL-36*α* can upregulate IL-17*α*, IL-23, and TNF-*α* expression, building a proinflammatory network with potentially pathogenic self-perpetuation in inflammation diseases. Additionally, the aforementioned cytokines in turn stimulate IL-36*α* production [39]. Due to dissimilarity in the constitution of inflammatory cells in autoimmune blistering diseases, it was important to examine the IL-36 immunoexpression in lesions. This cytokine may have an influence on enrollment of various cells to the skin. Frey et al. [40] showed that the novel cytokine IL-36*α* is upregulated in the synovial tissue in psoriasis and rheumatoid arthritis patients compared to osteoarthritis patients. In contrast, the receptor for IL-36*α* (IL-36R) as well as the natural antagonist of IL-36*α* (IL-36Ra) was equally expressed throughout all three arthropathies. Upregulation of IL36*α* is particularly linked to the presence of inflammatory infiltrates in the tissue. These findings suggest that IL-36*α* is a cytokine which is closely linked to inflammatory processes and expressed when leukocyte infiltration is observed in affected organs. Earlier data showing that IL-36*α* promotes the expression of proinflammatory cytokines further support this concept.

Our findings showed that IL-36*α* is overexpressed in autoimmune blistering diseases, especially in DH and PV and BP too. The high serum levels of IL-36*α* in all the study groups may be connected with activation of many cytokines responsible for influx in epidermis and dermis in these diseases.

Immunoexpression of IL-36*α* was shown in many of cells, for example, epidermal cells, lymphocytes, and mononuclear cells. That is why in these diseases, the serum levels may be higher in the control group. In the development of some inflammatory skin disorders with the release of interleukins from epidermal cells, one of these, IL1*α*, has an important role. Th17 cells, involved in autoimmunity and inflammation, can emit interleukins 17 and 22 in reply to IL-1*α* stimulation, and we have shown the increased level of IL-17 in autoimmune blistering diseases as well [9, 11, 33, 34, 37].

Unfortunately, there are still single papers supporting the role of IL-36*α* in the development of blistering conditions. In our examinations, IL-36*α* immunoexpression was higher in DH patients than in PV and BP groups. Pathological lesions in examined diseases are associated with neutrophilic and eosinophilic influx [41]. Some studies examined the influence of different factors on eosinophilic infiltration. In our opinion, various chemokines may take part in eosinophilic activation in bullous pemphigoid, and that is why, we showed the difference in serum level of IL36 in these diseases.

Studies by Gabay and Towne [42] have shown that IL-36 induces the production of IL-6 by fibroblasts. A study by Frey et al. [3] extended this notion by showing that IL-36*α* elicits a significant upregulation of interleukins 6 and 8 in fibroblasts. This represents a major proinflammatory trigger since IL-6 stimulates the synthesis of TNF-*α* and IL-8 acting as a major chemoattractant molecule for further leukocyte migration.

In autoimmune blistering diseases apart from the antigen-antibody reaction, the influx of inflammatory cells is essential for the appearance of bullae. Cytokines such as IL-6 and IL-8 have shown to be involved in the pathological process of a murine model of BP [19, 43].

Abnormality in the regulation of cytokine signaling may result in the pathogenesis of autoimmune conditions. Proinflammatory agents (e.g., IL-1, IL-6, and TNF-*α*) appear to have a substantial role in the control of autoimmunity, and overproduction of cytokines can provoke some autoimmune disorders [44]. Levels of proinflammatory cytokines have been examined, for example, in rheumatoid arthritis [45], vitiligo [46], lichen planus [47] and psoriasis [48], bullous pemphigoid, and dermatitis herpetiformis [16, 49]. Though various studies have estimated the proinflammatory cytokine concentrations in PV patients, their results are controversial due to the small group of examined patients [50].

Interleukin 6 is a strong agent to change immunological reply from the induction of Foxp3 T cells to Th17 cells [51]. Raised IL-6 serum concentrations in PV patients were first confirmed in D'Auria et al.'s study [52]. They also showed that IL-6 concentration was correlated with the number of blisters and was remarkably reduced after treatment.

Elevated concentration of IL-6 in the sera of PV patients was announced in the active phase and in the remission of pemphigus in Narbutt et al.'s study [50].

IL-8 has an important role in the acute phase of the disorder by enrolling and stimulating neutrophilic cells. D'Auria et al. demonstrate the concentration of IL-8 was noticeable in 6 of 25 PV individuals without any significant difference with control groups [52]. However, Keskin et al. [53] indicated a raised concentration of interleukin 8 in a study on a small PV group. The incompatibility could emanate from the difference in kits used to estimate the concentrations of IL-8.

Chu et al. [54] confirmed the immunoexpression and proinflammatory activity of IL-36 in patients with lupus erythematosus. Serum levels of IL-36*α* and *γ* and the ratio of circulating IL-36R-positive B cells in total B cells were remarkably elevated in the active stage of the disease compared with that of the control group. IL-36*α* and IL-36*γ* correlated positively with elevated IL-10 concentration and disease activity. IL-36*α* has a considerable effect on SLE patients via elevated production of IL-6 and CXCL8. This investigation showed that overexpression of this cytokine may influence on pathophysiology of SLE. A group of interleukin 36 is expressed strongly in dermis, for example, in psoriasis, and the antagonists of IL-36Ra can reduce uncontrolled inflammation. The possible action of interleukin 36 in rheumatoid arthritis and Crohn's disease is still under discussion [42].

Various cell types in skin, synovium and intestinal mucosa, can produce interleukin 36. In cultures of mononuclear cells, IL-36*β* and IL-36Ra were produced constitutively but interleukin 36*α* and *γ* after lipopolysaccharide stimulation [45].

In our results, we confirmed that IL36*α* level is elevated only in examined groups, with the highest level in DH patients. Although the positive correlation between IL-36*α* and antibody titers that we have shown only in the DH group, IL-36*α* level can be a marker of inflammation and disease activity in our opinion. It is probably connected with dominated neutrophilic influx in the involved skin in DH.

As we can notice, IL-36 family members as other IL-1 superfamily proteins need N-terminal cleavage to reach the entire biological activity. The mechanisms of IL-36Ra activation steel are weakly known. From all of the enzymes, produced by neutrophils, only elastase can cleave IL-36Ra, transforming it to a highly active antagonistic type [55].

Protease, of neutrophil origin, blocks IL36-induced cytokine production by activating IL-36Ra [20] and diminishes inflammatory cell influx following cytokine production. These studies firmly confirm that elastase of neutrophil origin plays a crucial role in the function of IL36Ra and shows that neutrophils can act a regulatory role in psoriatic skin, balancing the IL-36 activity [55].

One of paths can concern the neutrophil skin influx, link to the IgA with Fc*α*RI, and emit proteases that destruct the dermal-epidermal junction, leading to creation of vesicles in DH [56]. Marietta et al.'s [56] discovery of neutrophil elastase localized within the epidermis would promote this hypothesis. The factors that enroll and initiate neutrophils within these sites of immunoglobulin A deposition are undisclosed [57], but IL-36 may be involved in this mechanism.

In other study [6], concentration of IL-36 was elevated remarkably in the serum and salivary glands of Sjögren's syndrome patients. Serum levels of IL-36a were correlated with disease activity, indicating a possible function of this cytokine in controlling the activation of the immune network in Sjögren's syndrome. Action of antibodies in bullous pemphigoid and dermatitis herpetiformis cannot completely elucidate some valid features of the syndrome like the problem with transferring with the antibodies or the presence of lesional influx of inflammation cells that is obligatory for disorder initiation. Albeit there are a lot of immune mechanisms, links between them in blistering diseases have not been well characterized [58].

The supposition is that interleukins 36 and 17 have a significant role in blistering diseases and can characterize features of the disorder not excused by the autoimmune theory. In our study, we failed to show a correlation between the level of IL-36 and the level of antibodies in DH and between IL-17 and IL-36 levels. Perhaps, only in the case of dominant neutrophilic influx can correlations between IL-36 and IL-17 levels and disease activity be searched.

Many evidences suggest that activation of the interleukin-17-releasing cells could be related to the neutrophilic response and the progress of serious types of inflammation diseases, for example, asthma [59]. In reply to some immunological processes in the skin, change in innate immune responses initiates different inflammatory processes that may establish the identity of the initial IL-17-producing cells in the DH.

The last studies have shown the interleukin-1 family cytokines, IL-36*α*, IL-36*β*, and IL-36*γ*, as the crucial factors of dermis inflammation. IL-36 family is produced as inert precursors and needs enzymatic transformation for activation. Some papers show that interleukin 36 is activated, for example, by the neutrophil-derived proteases increasing their biological function. These data can confirm how neutrophils may increase inflammatory response [60].

Probably, that is why we can confirm a positive correlation between IL-36 and IL-17 and the level of anti-TG antibodies in DH, but not in BP and PV. Anti-TG antibody titer correlates with the activity of disorder and neutrophilic influx in the skin in DH patients.

This cytokine IL-36 can be a value in the sera of patients and can be used as a marker of the activity of disorder and remission because in all autoimmune bullous diseases, concentration of IL-36 is higher in the control group. The data received could also lead to the progress in new therapeutic options for this and other blistering diseases.

## 6. Conclusions

There are single data that contribute to the understanding of the contribution of IL-36 in the development of blistering diseases. According to our results, IL-36 concentration in sera was higher in patients with DH as compared to PV and BP patients. Though this distinction in serum level between DH and PV patients was less than between DH and BP and healthy subjects, this cytokine can play an essential role in pathogenesis of aforementioned diseases. This data could also help in the advancement of treatment for those and other autoimmune blistering diseases.
